# Kitchen Characteristics and Practices Associated with Increased PM_2.5_ Concentration Levels in Zimbabwean Rural Households

**DOI:** 10.3390/ijerph20105811

**Published:** 2023-05-12

**Authors:** Shamiso Muteti-Fana, Jafta Nkosana, Rajen N. Naidoo

**Affiliations:** 1 Discipline of Occupational and Environmental Health, School of Nursing and Public Health, Howard College Campus, University of KwaZulu Natal, Durban 4041, South Africa; 2 Unit of Family Medicine, Global and Public Health, Department of Primary Care Sciences, Faculty of Medicine and Health Sciences, University of Zimbabwe, 3rd Floor, Parirenyatwa Hospital Grounds, Harare P.O. Box A178, Zimbabwe

**Keywords:** biomass fuels, fine particulate matter (PM_2.5_), household air pollution, kitchen characteristics, women, Zimbabwe

## Abstract

Household air pollution (HAP) from biomass fuels significantly contributes to cardio-respiratory morbidity and premature mortality globally. Particulate matter (PM), one of the pollutants generated, remains the most accurate indicator of household air pollution. Determining indoor air concentration levels and factors influencing these levels at the household level is of prime importance, as it objectively guides efforts to reduce household air pollution. This paper describes household factors associated with increased PM_2.5_ levels in Zimbabwean rural household kitchens. Our HAP and lung health in women study enrolled 790 women in rural and urban households in Zimbabwe between March 2018 and December 2019. Here, we report data from 148 rural households using solid fuel as the primary source of fuel for cooking and heating and where indoor air samples were collected. Data on kitchen characteristics and practices were collected cross-sectionally using an indoor walk-through survey and a modified interviewer-administered questionnaire. An Air metrics miniVol Sampler was utilized to collect PM_2.5_ samples from the 148 kitchens over a 24 h period. To identify the kitchen features and practices that would likely influence PM_2.5_ concentration levels, we applied a multiple linear regression model. The measured PM_2.5_ ranged from 1.35 μg/m^3^ to 1940 μg/m^3^ (IQR: 52.1–472). The PM_2.5_ concentration levels in traditional kitchens significantly varied from the townhouse type kitchens, with the median for each kitchen being 291.7 μg/m^3^ (IQR: 97.2–472.2) and 1.35 μg/m^3^ (IQR: 1.3–97.2), respectively. The use of wood mixed with other forms of biomass was found to have a statistically significant association (*p* < 0.001) with increased levels of PM_2.5_ concentration. In addition, cooking indoors was strongly associated with higher PM_2.5_ concentrations (*p* = 0.012). Presence of smoke deposits on walls and roofs of the kitchens was significantly associated with increased PM_2.5_ concentration levels (*p* = 0.044). The study found that kitchen type, energy type, cooking place, and smoke deposits were significant predictors of increased PM_2_._5_ concentrations in the rural households. Concentrations of PM_2.5_ were high as compared to WHO recommended exposure limits for PM_2.5_. Our findings highlight the importance of addressing kitchen characteristics and practices associated with elevated PM_2.5_ concentrations in settings where resources are limited and switching to cleaner fuels may not be an immediate feasible option.

## 1. Introduction

An estimated 2.6 billion people worldwide are at risk of ill health and premature deaths annually due to household air pollution (HAP) from domestic use of biomass fuel [1] (wood, manure, crop wastes, coal, charcoal, and cow dung) for cooking, heating, and lighting [1]. HAP was associated with 3.2 million deaths in the year 2020, with low- and middle-income countries (LMICs) being disproportionately affected [2]. Most people who use biomass as their primary source of fuel live in rural areas [3], and close to 700 million [4] of them live in Africa, predominantly in sub-Saharan Africa [5,6]. Roughly 60% of the burden of disease related to HAP is borne by women, who have increased exposure to HAP due to gender-based domestic roles [7,8].

Inefficient burning cookstoves used with biomass fuels generate a wide range of air pollutants [9], including particulate matter (PM) [10]. The levels of pollutants that household members are exposed to daily when cooking with solid fuels over an open fire or on inefficient stoves are midway between those of passive smoking and active smoking [11]. PM is responsible for some of the most harmful impacts of biomass fuel exposure on its own [12,13]. The concentration of PM of diameter ≤ 2.5 μm (PM_2.5_) is an important metric to estimate the risk of respiratory illness since these very small particles have the capacity to penetrate deep into the lungs when inhaled [14]. The World Health Organization (WHO) has recommended exposure threshold limits of 15 μg/m^3^ 24 h mean for PM_2.5_ [15].

Indoor concentrations of pollutants are affected by several household-level factors [9], which may vary between places. Understanding of these factors in various environments crucial, as some of them can be modified to reduce indoor air pollution levels. Typically, these characteristics are context-dependent, as they are influenced by economic conditions and cultural differences. Previous research has examined the household characteristics associated with PM_2.5_ concentration levels in diverse environments [16,17,18]. Pollard et al. (2014), after multivariate analysis, found that thatched roof and hours spent cooking were positively associated with increased 24 h PM_2.5_ concentrations, whilst the presence of a chimney did not [19]. Shupler et al. (2022) carried out a multinational study in which they predicted household and personal exposure to PM_2.5_ and concluded that stove type, heating fuel, and presence of a chimney partially explained the variation of PM_2.5_ kitchen concentrations [20]. In all these various studies, context played a major role in the indoor concentration levels. Clark et al. (2010) found a link between the concentration levels and the wall type [21]. Another study in Uganda by Nakora et al. (2020) concluded that kitchens with mud and concrete walls wear out faster than those with other types of material, creating dust particles and thereby causing high PM_2.5_ concentrations [22]. In their study, Jafta et al. (2017) emphasized the contribution of low-quality building materials to indoor air pollution by dispersing particles into the air when disturbed [23]. From these studies, it is evident that there are contextual and cultural differences that affect HAP, which makes it difficult to generalize findings across and within countries. It is therefore important to understand the context-specific determinants of HAP in the Zimbabwean setting.

In Zimbabwe, about 67.7% of people live in rural regions [24], and mostly rely on biomass as their primary fuel source, putting a significant percentage at risk of effect of exposure to HAP. Recommendations from various studies have been to shift from dirty fuels to clean fuels [25,26,27]. However, currently, in the Zimbabwean context, this may not be feasible due to other social and public health priorities, such as malnutrition, overcrowding, poor housing conditions, and poverty. Given the probability that the use of biomass is likely to continue, it is important to understand the exposure patterns to HAP in order to design and implement context-specific interventions. Contextual differences in kitchen characteristics and cooking practices can result in a wide range of HAP concentrations, hence the need to carry out studies to evaluate exposures across various settings [10]. To accurately estimate exposure to PM_2.5_, it is necessary to understand a number of factors at the household level [9], including the type of fuel used, the design and location of the kitchen, and the type of stove. These will enable mechanisms to plan, implement, and evaluate preventative programs. The aim of this paper was to identify the kitchen characteristics and household practices that influence PM_2.5_ concentration levels in rural Zimbabwean households.

## 2. Materials and Methods

### 2.1. Setting and Study Design

In the rural district of Goromonzi in the Mashonaland East province of Zimbabwe, a community-based, cross-sectional study was conducted among women aged 18 years and older who use biomass as their primary source of fuel. We evaluated the 24 h kitchen area concentrations of PM_2.5_ in 148 of the 790 households recruited for the ***Biomass smoke and lung health in adult women in Zimbabwean families study.***

**Figure 1:**
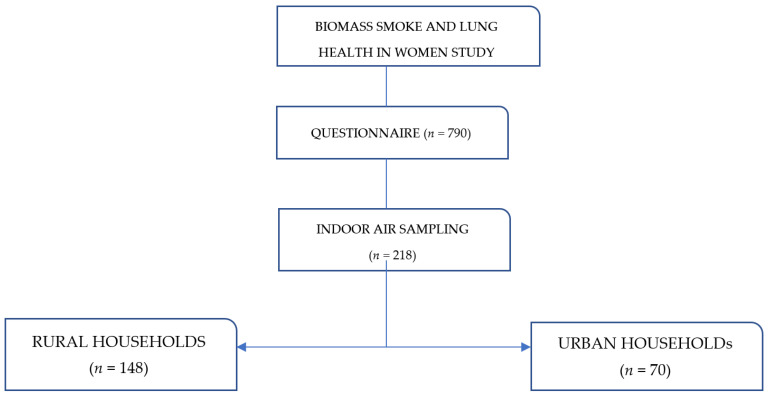
Flow diagram of the recruitment process.

The district directory of enumeration areas of Zimbabwe was used as the sampling frame to randomly select Goromonzi as the study area. The villages were selected using a multistage stratified sampling design to obtain a representative sample. At each household, we chose one woman who was 18 years old or above to aid with the walkthrough survey and respond to the questionnaire on kitchen characteristics. The woman who headed the household and did most of the cooking in the homestead was chosen when there were many women in a household who matched the inclusion criteria. All rural households (*n* = 466 out of the 790 households in the parent study) were using biomass as their primary source of fuel. However, for this paper, we selected only those households (*n* = 148) where indoor air samples had been collected (see Figure 1). Data were collected between March 2018 and December 2019. All air samples were collected during the hot/summer season.

### 2.2. Environmental Exposure Assessment

#### 2.2.1. Indoor Walkthrough Assessment and Questionnaire Administration

The walkthrough assessment involved observing and recording the kitchen characteristics including ventilation (presence or absence of windows, presence, or absence of a chimney), construction material used (type of roof, type of floor, and wall material), as well as evidence of exposure to biomass smoke (residue on surfaces with categories such as heavy—dark smoke deposits with black visible soot substances with some hanging from the roof surfaces; moderate—lightly stained surfaces which evidently shows accumulation of smoke particles; or none—surfaces that have not been stained by smoke). Kitchen types were categorized into traditional (thatched kitchens) and townhouse (brick under asbestos/tile/corrugated iron kitchens), while cooking areas were categorized as separate kitchen indoors and hut/detached building and outdoors. We further categorized stoves as traditional solid fuel stoves and improved solid fuel stoves. For this study, a traditional solid fuel stove is one with an open fire and no smoke exhaust. An improved solid fuel stove is one that has an exhaust, such as a chimney or a hole in the side of the stove, which channels smoke outside of the room. 

#### 2.2.2. Fine Particulate Matter (PM_2.5_) Sample Collection

The primary use of solid fuel for cooking was used as an indicator of HAP exposure among rural participants. We measured the indoor (kitchen area) concentration of airborne particulate matter with an aerodynamic diameter < 2.5 µm (PM_2.5_) over a 24 h period continuously using a calibrated battery-operated Air Metrics Minivol model TAS 5.0 portable air sampler equipped with a 47 mm PTFE (Teflon/polytetrafluoroethylene) filter with 2.0 μm pore size [28,29]. Calibration of the sampler flowrate was performed at the start and at the end of each sampling period using a rotameter. The sampling equipment was placed in the kitchen about 1.5 m high to simulate the breathing zone and at least 1.0 m away from the wall. The air samplers were operated at a flowrate of 5 L/min as per manufacturer guidelines. At the end of the sampling period (24 h), the filters were carefully removed from the samplers, put back into filter cassettes, and transported back to the laboratory for analysis. Sampling results were reported in micrograms/cubic meter. A field blank filter was included for each batch of filters used to sample PM_2.5_, and we analyzed gravimetrically as part of quality control. We recorded activities done during the sampling period at the end of the sampling time to determine any other possible sources of PM_2.5_ during the sampling period.

### 2.3. Gravimetric Analysis

Weighing of filters was done using a micrometric balance (Sartorius entris Ag, Cole Parmer, Wertheim am Main, Germany) with accuracy of 0.001 mg in a laboratory with controlled humidity of 36% to 41% and temperature of 18–23 °C. Prior to the pre-sampling and post-sampling weighing sessions, all filters were equilibrated for 24 h. The filters were pre-weighed until the 0.01st milligram was stable according to the American Society for Testing and Materials (ASTM) D 1739. Filters were then stored in filter cassettes in a box and transported to the field at room temperature. The flow rate of the sampler was calibrated during deployment, and at the conclusion of the sampling time, the flow rate was once more measured using a rotameter. The change in mass at the end of the sampling period minus the blank mass change was simply divided by the volume of air sampled, and a concentration level was produced, as indicated in Equation (1). The measured PM_2.5_ concentration in μg/m^3^ was calculated according to the following equation:

(1) $$C=\frac{\left(M_{2}-M_{1}-B\right)}{V_{s}}$$

where:

C = PM_2.5_ concentration levels in mg/m^3^;
M_1_ = filter mass before sampling (mg);
M_2_ = filter mass after sampling (mg);
B = average mass change of blanks (mg);
V_S_ = volume of air sampled (m^3^) [30].

It was calculated using the typical sample flow rate, the sampling duration, and the surrounding pressure and temperature. As a result, each household had its air tested for about 7200 L (7.2 m^3^) throughout the course of a 24 h period. The ultimate mass concentration was estimated in mg/m^3^. The limit of detection (LOD) of the weighing procedure was determined from three times the standard deviation of the weight changes of field blank filters [31]. We used a total of 11 blank filters in computing the limit of detection at 2.71 μg/m^3^ [32]. We then substituted left-censored data with LOD/2, which is 1.35 μg/m^3^.

### 2.4. Statistical Methods 

The statistical data analysis was conducted in R statistical computing software version 3.6.3. The descriptive statistics were presented in the form of counts and percentages for categorical variables whereas the numeric variables displayed as minimum, maximum, and quartiles. The PM_2.5_ distribution was highly skewed such that either the Wilcoxon [33] or Kruskal–Wallis test [34] was used to assess the difference in the PM_2.5_ levels between the categories of the variables. Multiple linear regression was fitted on the log-transformed PM_2.5_. Stepwise regression was used to determine the predictors of the PM_2.5_ levels. All the tests were conducted at 5% level of significance. 

## 3. Results

Samples from 148 kitchens that used biomass as their primary source of fuel were analyzed for this work. The median PM_2.5_ concentrations in the 148 houses was 278 μg/m^3^ (IQR: 52.1, 472).

### 3.1. Kitchen Characteristics and PM_2.5_

Table 1 below shows that most participants (92.6%) had traditional kitchens as opposed to townhouse kitchens. There was a statistically significant difference in PM_2.5_ concentration between traditional kitchens and townhouse-type kitchens, with median concentration levels being 291.7 μg/m^3^ (IQR = 97.2–472.2 μg/m^3^) and 1.35 μg/m^3^ (IQR = 1.3–97.2 μg/m^3^), respectively (*p* = 0.008). In addition, it was observed that kitchens that had mud walls had increased PM_2.5_ concentration levels compared to kitchens that had walls made of tile, asbestos, or corrugated iron (*p* = 0.134). However, this discrepancy did not reach statistical significance. A limited number of kitchens (22.3%) included in the study were found to have chimneys. These kitchens exhibited elevated levels of PM_2.5_ concentration in comparison to those without chimneys (*p* = 0.102). However, the observed difference was not statistically significant. The study findings indicate that a minority of the participants (38.4%) reported preparing meals more than three times a day, and their kitchens had lower PM_2.5_ concentration levels compared to those who prepared less than three meals per day, but the observed difference was not statistically significant (*p* = 0.100).

Construction year of the kitchen (pre or post 1985 (*p* = 0.286), roof type (*p* = 0.618), floor type (*p* = 0.944), having ever stayed with a smoker (*p* = 0.656), biomass use duration (*p* = 0.898), time spent cooking per day (*p* = 0.902), window opening frequency (*p* = 0.944), and monthly income (*p* = 0.966) had no statistically significant associations with the PM_2.5_ concentration levels in the sampled households (Table 1). 

### 3.2. Sources of PM_2.5_ Levels

Evidence of tobacco smoking was reported by few participants (4.7%); however, it was significantly associated with an increase in PM_2.5_ concentration levels, with a median concentration level of 597.2 μg/m^3^ (IQR of 326.4–1319.4) compared to 263.9 μg/m^3^ (IQR (1.3–97.2) in the kitchens of those who did not report it. The most used type of stove in this study was the traditional solid fuel stove (87.8%), and its use was significantly associated with increased PM_2.5_ concentration levels as compared to the improved solid fuel stove (*p* = 0.072). Most of the participants in this study (87.8%) used wood in combination with other biomass fuels to enhance the fire during cooking times. Comparing use of wood only and use of wood mixed with other types of biomass fuels such as cow dung and crop residues, using wood only was associated with decreased PM_2.5_ concentration levels (*p* = 0.001). Most participants (70.3%) reported cooking inside the hut, while the rest of the respondents reported that they cooked outdoors or in other separate rooms. Cooking indoors was significantly associated with increased PM_2.5_ concentration levels, with the median being 333.3 μg/m^3^ compared to 118.1 μg/m^3^ among those who did not cook indoors. More than half (53.4%) of the kitchens that were sampled had heavy smoke deposits on the walls and roof. These kitchens were significantly associated with increased PM_2.5_ levels (*p* = 0.018) as compared to the kitchen that had none to modest smoke deposits.

Scented candles (*p* = 0.786), incense use (*p* = 0.263), and use of a space heater (*p* = 0.632) had no significant association with PM_2.5_ levels in the sampled households (Table 2).

### 3.3. Multiple Linear Regression Model with PM Concentrations as Dependent Variables

Table 3 presents the outcomes of a multiple regression analysis that was conducted to model the relationship between PM_2.5_ and the variables related to kitchen characteristics and exposure. The variables included in the model were those with a *p*-value below 0.25 in the univariate analysis. The statistical analysis revealed a significant association (*p* = 0.033) between the traditional kitchen type and elevated levels of PM_2.5_ in comparison to the townhouse kitchen type. The study also found a significant correlation between increased levels of PM_2.5_ concentration and the energy type, cooking in a hut, and heavy smoke deposits. In addition, the use of wood in combination with other forms of biomass such as cow dung and crop residue were found to be correlated with heightened levels of PM_2.5_. 

Contrary to the univariate analysis findings, use of traditional solid fuel stoves was associated with decreased PM_2.5_ concentration levels. Preparing meals more than three times was found to have a statistically significant correlation with low PM_2.5_ concentration levels (*p* = 0.054). Compared to households that had none to modest smoke deposits on the kitchen roofs and windows, heavy smoke deposits in the kitchen were associated with increased PM_2.5_ concentration levels, and the difference was statistically significant (*p* = 0.043). There was a positive correlation between households that had evidence of tobacco smoking and elevated levels of PM_2.5_ (*p* = 0.056), but it was not statistically significant. Having a chimney (*p* = 0.622) and cement/wood/corrugated iron wall type (*p* = 0.251) were not associated with the PM_2.5_ concentration levels.

### 3.4. Predictive Multiple Linear Regression Modelling with Strongest Predictors

Table 4 displays the primary factors influencing PM_2.5_ concentration levels as determined by the stepwise multiple linear regression model. The statistical model exhibited a superior fit, as evidenced by a significant overall goodness-of-fit (*p*-value < 0.001) and an adjusted R^2^ value of 0.355. The findings of the study indicate a statistically significant association between traditional kitchens and elevated levels of PM_2.5_ as compared to townhouse kitchens (*p* = 0.033). Statistically significant correlations were also noted between elevated levels of PM_2.5_ concentration levels and energy type (*p* = 0.001), cooking in a hut (*p* = 0.001), and heavy smoke deposits (*p* = 0.044). Furthermore, the use of wood in conjunction with other types of biomass such as cow dung and crop residue was associated with increased PM_2.5_ concentration (*p* = 0.001) compared to the use of wood only. 

Whilst there was a positive correlation between households with evidence of tobacco smoking and elevated levels of PM_2.5_ (*p* = 0.056), the relationship was not statistically significant. Even though use of traditional solid fuel stove was initially found to be associated with increased PM_2.5_, it became significantly associated with reduced PM_2.5_ concentration levels after the multivariate analysis (*p* = 0.006). Comparing the number of meals prepared per day, those who prepared more than three meals per day had decreased PM_2.5_ concentration levels compared to those who prepared less than three meals per day; however, the difference was not statistically significant (*p* = 0.056).

(2) $$\begin{array}{ll} E\left\{\log\left({\mathrm{PM}}_{2.5}\right)\right\}= & \beta_{k1}I\left(\mathrm{Kitehen}\;\mathrm{type}\;=\;\mathrm{Traditional}\right)+{\;\beta }_{k2}I\left(\mathrm{Evidence}\;\mathrm{of}\;\mathrm{tobacco}\;\mathrm{smoke}=\;\mathrm{Yes}\right)\\ & +{\;\beta }_{k4}I\left(\mathrm{Stove}\_\mathrm{type}=\;\mathrm{Traditional}\;\mathrm{solid}\;\mathrm{fuel}\;\mathrm{stove}/\mathrm{Open}\;\mathrm{fire}/\mathrm{Other}\right)\\ & +{\;\beta }_{s1}I\left(\mathrm{Energy}\_\mathrm{type}=\;\mathrm{Wood}/\mathrm{Other}\right)+{\;\beta }_{s2}I\left(\mathrm{Cooking}\;\mathrm{place}=\;\mathrm{Indoors}\right)\\ & +{\;\beta }_{s3}I\left(\mathrm{Food}\_\mathrm{preparation}\_\mathrm{frequency}\;=3+\right)+{\;\beta }_{s5}I\left(\mathrm{Smoke}\;\mathrm{deposits}\;=\;\mathrm{Heavy}\right) \end{array}$$

## 4. Discussion

Our study showed that PM_2.5_ concentration levels in Zimbabwean rural households are high, with most of them having levels above the WHO-recommended exposure limits for PM_2.5_. This is congruent with previous studies done in Zimbabwe, where levels of >40 μg/m^3^ have been reported [36,37,38], and elsewhere, including South Africa [10,16,17,18,23,39]. The factors that proved to be the best predictors of PM_2.5_ concentration after stepwise regression model were kitchen type, energy type, cooking place, and smoke deposits. 

Consistent with previous studies, traditional type of kitchens had significantly higher levels of PM_2.5_ [16]. These kitchens are usually made of poles, mud, and grass and have thatched roofs, which have a higher likelihood of accumulating indoor contaminants [17]. It is also important to note that most respondents with traditional-style kitchens were in the low socio-economic group earning below USD 150 monthly even though income was not statistically significantly associated with PM_2.5_ concentration levels in the univariate analysis. The energy ladder depicts socioeconomic status as an important determinant of fuel preferences at the household level [40,41,42]. 

Additionally, income influences other factors such as kitchen characteristics, stove type, and food preparation frequency. Fandiño-Del-Rio et al. (2020) also reported that higher kitchen PM concentrations were likewise related to lower levels of wealth in Peru [17]. The assumption is that low-income households are more likely to utilize biomass and other inexpensive, dirty fuels since they are less likely to be able to afford alternative, cleaner fuels. Additionally, with fewer resources, it is possible that the kitchen’s design and construction materials are of poor quality, which encourages the buildup of pollutants indoors [23]. In a study examining the patterns and predictors of indoor air pollution in India, Baumgartner et al. (2021) found kitchen structure as one of the significant predictors of personal PM_2.5_ exposure among people who used biomass as the primary source of fuel [43].

A significant percentage of the study participants (88.4%) indicated that they used traditional solid fuel stoves. Contrary to what other studies have found, these households exhibited lower PM_2.5_ concentration levels compared to those households that use the improved solid fuel stoves. This finding is opposed to what Clark et al. (2016) found, where improved cooking stoves were associated with reduced indoor air concentration levels [21]. However, the study also highlighted the limitation of using the type of stove to assign exposure, as there is a great deal of variation in different stove types and levels of PM_2.5_ concentrations associated with each type [21]. Yip et al. in Kenya also found that the mean 48 h concentrations of PM_2.5_ were lower in improved solid fuel stoves than in traditional solid fuel stoves [44]. 

When compared to the use of wood alone, the use of wood combined with other types of biomass such as cow dung and crop residues resulted in a significant increase in PM_2.5_ concentration levels. This could be because the other types of biomass are regarded as the least-efficient burning types of biomass fuels on the energy ladder [41]. Research has highlighted that most solid fuel users do not utilize a single type of fuel but rather a mixture of multiple types depending on availability [25]. It is therefore appropriate to urge rural communities such as the one that was included in our study to use wood as their only fuel source in situations where there are no other cleaner alternatives. 

It is well known that using biomass fuels indoors for cooking causes HAP, especially when the area is not well ventilated. Throughout this study, we detected higher PM_2.5_ concentrations in kitchens where cooking occurred inside. When cooking is done inside, the released smoke remains suspended in the atmosphere for prolonged periods of time mainly due to poor ventilation, exposing residents to home air pollution. The element of cooking inside is connected to kitchen type since it determines whether or not indoor air pollutants will accumulate; it all relies on the type of the kitchen.

In this investigation, there were smoke deposits in around 53.4% of the kitchens, and this was statistically significant. It is widely known that higher PM_2.5_ concentration levels are linked to the presence of smoke deposits in the kitchen or cooking area. Previous studies have highlighted how certain contaminants are deposited on kitchen walls before leaving the kitchens [45]. This phenomenon, when it occurs more often, continues to leave dark marks on the roof and walls, and this is a common scenario in the majority of rural homes: some particulate matter is retained on the kitchen walls or roof rather than escaping outside [45,46].

Approximately 38.4% of the study participants reported that they prepared more than three meals per day, and their kitchens recorded lower PM_2.5_ concentration levels compared to those who prepared less than three meals per day. It may be difficult to use this variable as a proxy for indoor PM_2.5_ concentration levels unless there are more details about the types of meals that were prepared and the duration it took to prepare each meal. There are many contextual differences in the way that meals are prepared in one household or another. Therefore, it is difficult to interpret the association between the measured PM_2.5_ concentration levels and the number of prepared meals per day. A person may report making one or two meals per day but may spend more time doing so than someone who prepares more than three meals per day.

### Strengths and Limitations of the Study

While previous studies have demonstrated that monitoring both indoor and outdoor air sampling is necessary for characterizing HAP, such monitoring was not possible in this investigation due limited resources and logistical constraints. However, some studies have argued that the background ambient PM_2.5_ concentrations in rural areas, as in the case of our context, are unlikely to significantly contribute to the exposures experienced by individuals who cook with biomass [19], while the case maybe different in urban areas, where there is a great deal of traffic and other industrial activities that generate particulate matter that infiltrates indoors. Another study by Shupler et al. (2022) indicated that ambient air pollution influences HAP exposure though sometimes it may not be feasible to quantify the effect of ambient air on HAP [20]. In a different context, in India, Anand et al. (2021) concluded that ambient PM_2.5_ was the most significant predictor for indoor concentration levels [47] such that with adequate conditions, polluted air can infiltrate indoors. The infiltration of contaminants from ambient air into interior spaces has been documented in most industrialized countries when the ratio of indoor to outside pollution is less than one [48]. The majority of developing countries, on the other hand, have discovered that interior air pollution levels were much higher than outdoor air pollution levels, especially in rural areas where the indoor/outdoor ratio is greater than one, and pollutant exfiltration from inside to outside is projected [45].

However, there are important strengths in our study; for instance, we used the gravimetric sampling technique, which is considered the gold standard in air sampling because it is precise and accurate. Furthermore, we were able to collect detailed data from the households and carried out indoor walk-through assessments to aid with gathering adequate information about the households.

## 5. Conclusions and Recommendations

When compared to the WHO-recommended exposure limits for PM_2.5_, the PM_2.5_ concentrations in this study were excessive. Significant predictors of PM_2.5_ concentrations included kitchen type, energy type, cooking place, and smoke deposits. Our findings underscore the need to educate the public on the impact of HAP exposure and the best practices for minimizing exposure in the home as a means of lowering pollution levels. A coordinated effort including all stakeholders might produce encouraging outcomes. An acceptable strategy would include a nationwide public awareness campaign on the link between indoor air pollution and poor health and practical action. It is important to look at how the traditional kitchens are designed and aim to improve kitchen design to reduce indoor accumulation of smoke. There is a need to instill behavior change among the at-risk population by adopting low-cost methods to prevent HAP. Policymakers can also play a role by enacting measures to ensure that kitchens are built to a certain standard using materials that may not promote accumulation of pollutants indoors. To reduce the indoor PM_2.5_ levels in rural kitchens in Zimbabwe, alterations to the kitchen’s physical characteristics to ensure that it allows air exchange during cooking times may be of benefit. 

## Figures and Tables

**Table 1:** PM_2.5_ and kitchen characteristics and lifestyle for rural participants in 148 households.

Characteristic	*n* (%)	PM_2.5_ Concentration Median (IQR)	*p*-Value
**Kitchen type**			
Townhouse	11 (7.4%)	1.3 (1.3, 97.2)	0.008 *
Traditional	137 (92.6%)	291.7 (97.2, 472.2)	
**Construction year ϕ**			
Pre 1985	26 (20.5%)	305.6 (138.9, 649.3)	0.286
Post 1985	101 (79.5%)	263.9 (27.8, 430.6)	
**Roof type**			
Thatch	102 (68.9%)	291.7 (114.6, 430.6)	0.618
Tile/Asbestos/Corrugated iron	46 (31.1%)	215.3 (27.8, 677.1)	
**Wall type**			
Mud	71 (48.0%)	291.7 (152.8, 500)	0.134
Cement/Wood/Corrugated iron	77 (52.0%)	250 (27.8, 430.6)	
**Floor type**			
Cement	136 (91.9%)	277.8 (55.6, 472.2)	0.944
Wood floor/Tile/Other	12 (8.1%)	298.6 (1.3, 506.9)	
**Chimney**			
No	115 (77.7%)	291.7 (118.1, 468.7)	0.102
Yes	33 (22.3%)	111.1 (27.8, 513.9)	
**Ever stayed with a smoker**			
No	70 (47.3%)	256.9 (55.6, 468.7)	0.656
Yes	78 (52.7%)	291.7 (55.6, 501.7)	
**Biomass fuel use duration ϕ**			
≤30 years	66 (45.2%)	284.7 (27.8, 451.4)	0.898
>30 years	80 (54.8%)	263.9 (97.2, 531.3)	
**Time spent cooking/day ϕ**			
<2 h	32 (22.2%)	263.9 (131.9, 395.8)	0.902
≥2 h	112 (77.8%)	277.8 (27.8, 479.2)	
**Food preparation frequency/day ϕ**			
<3	90 (61.6%)	277.8 (114.6, 513.9)	0.100
3+	56 (38.4%)	270.8 (1.3, 430.6)	
**Window opening frequency**			
Most of the time	119 (80.4%)	277.8 (41.7, 486.1)	0.944
Never/occasionally	29 (19.6%)	263.9 (111.1, 388.9)	
**Monthly income**			
<$150	129 (87.2%)	278(52.1–472)	0.966
$150+	19 (12.8%)	148(1.35–1940)	

ϕ Variables with less than 148 responses. * *p*-value considered significant at <0.05.

**Table 2:** Household characteristics and indoor PM_2.5_ concentration in 148 households.

Characteristic	*n* (%)	PM_2.5_ Concentration Median (IQR)	*p*-Value ^&^
**Evidence of tobacco smoking**			
No	141 (95.3%)	263.9 (41.7, 458.3)	0.045 *
Yes	7 (4.7%)	597.2 (326.4, 1319.4)	
**Scented candles use ϕ**			
No	135 (92.5%)	277.8 (48.6, 472.2)	0.786
Yes	11 (7.5%)	291.7 (132.6, 458.3)	
**Incense use ϕ**			
No	141 (96.6%)	277.8 (55.6, 500)	0.263
Yes	5 (3.4%)	263.9 (1.3, 291.7)	
**Stove type**			
Traditional solid fuel stove	130 (87.8%)	277.8 (100.7, 472.2)	0.072
Improved solid fuel stove	18 (12.2%)	1.3 (1.3, 444.4)	
**Energy type ϕ**			
Wood only	17 (11.6%)	1.3 (1.3, 41.7)	<0.001 *
Wood and other	130 (88.4%)	291.7 (114.6, 500)	
**Cooking place**			
Outdoors	44 (29.7%)	118.1 (1.3, 277.8)	<0.001 *
Indoors	104 (70.3%)	333.3 (159.7, 531.3)	
**Ventilation method**			
Doors/Windows	146 (98.6%)	277.8 (45.1, 472.2)	#
Other	2 (1.4%)	284.7 (267.4, 302.1)	
**Visible smoke deposits**			
None to modest	69 (46.6%)	250 (27.8, 388.9)	0.018 *
Heavy	79 (53.4%)	347.2 (125, 534.7)	
**Space heater**			
No	143 (96.6%)	277.8 (48.6, 468.7)	0.632
Yes	5 (3.4%)	277.8 (208.3, 722.2)	

# The data were not enough for statistical inference; ϕ variables with missing data are therefore less than 148 responses; ^&^ Wilcoxon test [35]; * *p*-value considered significant at <0.05.

**Table 3:** Multiple linear regression model with PM concentrations as dependent variables.

Explanatory	β	95% CI	*p*-Value
Kitchen_typeTraditional	1.51	0.12 to 2.90	0.033 *
Wall_typeCement/Wood/Corrugated iron	−0.38	−1.03 to 0.27	0.251
ChimneyYes	0.21	−0.62 to 1.04	0.622
Evidence_of_tobacco_smokingYes	1.46	−0.04 to 2.96	0.056
Stove_typeTraditional solid fuel stove/Open fire/Other	−1.34	−2.31 to −0.37	0.007 *
Energy_typeWood/Other	2.05	1.00 to 3.09	0.001*
Cooking_placeIndoors	1.26	0.54 to 1.98	0.001 *
Food_preparation_frequency3+	−0.67	1.36 to 0.01	0.054 *
Smoke_depositsHeavy	0.66	0.02 to 1.30	0.043 *

* *p*-value considered significant at <0.05.

**Table 4:** Multiple linear regression model with the strongest predictors.

Explanatory	β	95% CI	*p*-Value
Kitchen_typeTraditional	1.55	0.29 to 2.82	0.016 *
Evidence_of_tobacco_smokingYes	1.46	−0.04 to 2.95	0.056
Stove_typeTraditional solid fuel stove/Open fire/Other	−1.36	−2.33 to −0.39	0.006 *
Energy_typeWood/Other	2.10	1.06 to 3.13	0.001 *
Cooking_placeIndoors	1.21	0.50 to 1.93	0.001 *
Food_preparation_frequency3+	−0.67	−1.35 to 0.02	0.056
Smoke_depositsHeavy	0.65	0.02 to 1.28	0.044 *

* *p*-value considered significant at <0.05. Note: Predictor variables were used in Equation (2) as follows.

## Data Availability

The data presented in this study are available on request from the corresponding author.
